# Renal association clinical practice guideline on Anaemia of Chronic Kidney Disease

**DOI:** 10.1186/s12882-017-0688-1

**Published:** 2017-11-30

**Authors:** Ashraf Mikhail, Christopher Brown, Jennifer Ann Williams, Vinod Mathrani, Rajesh Shrivastava, Jonathan Evans, Hayleigh Isaac, Sunil Bhandari

**Affiliations:** 1 Abertawe Bro Morgannwg University Health Board, Swansea, Wales, United Kingdom; 2 Aneurin Bevan University Health Board, Newport, Wales, United Kingdom; 3 Nottingham University Hospitals NHS Trust, Nottingham, England; 4 Patient Representative, c/o The Renal Association, Bristol, United Kingdom; 5 Hull & East Yorkshire Hospitals NHS Trust, Hull, England

## Abstract

Anaemia is a commonly diagnosed complication among patients suffering with chronic kidney disease. If left untreated, it may affect patient quality of life. There are several causes for anaemia in this patient population. As the kidney function deteriorates, together with medications and dietary restrictions, patients may develop iron deficiency, resulting in reduction of iron supply to the bone marrow (which is the body organ responsible for the production of different blood elements). Chronic kidney disease patients may not be able to utilise their own body’s iron stores effectively and hence, many patients, particularly those receiving haemodialysis, may require additional iron treatment, usually provided by infusion.

With further weakening of kidney function, patients with chronic kidney disease may need additional treatment with a substance called erythropoietin which drives the bone marrow to produce its own blood. This substance, which is naturally produced by the kidneys, becomes relatively deficient in patients with chronic kidney disease. Any patients will eventually require treatment with erythropoietin or similar products that are given by injection.

Over the last few years, several iron and erythropoietin products have been licensed for treating anaemia in chronic kidney disease patients. In addition, several publications discussed the benefits of each treatment and possible risks associated with long term treatment. The current guidelines provide advice to health care professionals on how to screen chronic kidney disease patients for anaemia, which patients to investigate for other causes of anaemia, when and how to treat patients with different medications, how to ensure safe prescribing of treatment and how to diagnose and manage complications associated with anaemia and the drugs used for its treatment.

## Introduction

This clinical practice guideline provides recommendations on the management of anaemia of chronic kidney disease (ACKD) and serves as an update of the 5th edition module published online in 2010. The recommendations in this update have been graded using the modified GRADE system to indicate both the strength of each recommendation (strong or weak) and level of evidence for the recommendation (A-D) [1, 2]. As in the previous module The Renal Association (RA) endorses the NICE Guideline for anaemia management in chronic kidney disease 2015 [3] and adopts in this guideline update the nomenclature for classifying CKD from the NICE Guideline for chronic kidney disease in adults 2014 [4].

This guideline update covers the management of anaemia in adults, children and young people with anaemia associated with CKD. While there is no universally accepted classification for categorising the population with anaemia of CKD by age, this guideline adopts the classification set out in NICE Guideline [3] defined as follows:

- children: 0–13 years
- young people: 14–17 years
- adults: 18 years and over

For this guideline update systematic literature searches were undertaken to identify all published clinical evidence relevant to the review questions. Databases were searched for all published papers between January 2009 and November 2016, using relevant medical subject headings, free-text terms and study-type filters where appropriate. All searches were conducted in MEDLINE, PUBMED, Embase, and The Cochrane Library. Data search used the following search terms:

- Anaemia and CKD
- Anaemia and dialysis
- Blood transfusion and dialysis
- Erythropoietin, EPO, ESA
- ESA Resistance
- Immunosuppression and anaemia
- Immunosuppression and EPO
- Immunosuppression and blood transfusion
- Iron deficiency
- Iron therapy
- Iron toxicity
- Pure red cell aplasia
- Anaemia and dialysis
- Renal anaemia
- Renal transplant and anaemia
- Renal transplant and blood transfusion
- Renal transplant and EPO

This guideline is an update on previous Renal Association guidelines published in November 2010. The search covered the period from January 2009 to November 2016. The previous guidelines covered the periods prior to the above dates. Articles not written in English were not assessed. Articles available in abstract forms; letters; case reports; editorials or review articles were also excluded. Articles were assessed for relevance to the guideline topic, eligibility for inclusion in the evidence base for that guideline and methodological quality.

Articles were considered of particular relevance if they were describing:

- Prospective randomised or quasi-randomised trials
- Controlled trials
- Meta-analysis of several trials
- Cochrane systematic reviews.

Where evidence was available from the above sources, recommendations were based on these publications. Where there was a lack of evidence from high-quality studies, recommendations were based on current consensus and that was made clear in the document:

We also reviewed all related guidelines including those listed below:

- European Renal Best Practice (ERBP) for Anaemia in CKD [5, 6]
- Kidney Disease Outcomes Quality Initiative (KDOQI) Guidelines for Management of anaemia in CKD [7],
- Kidney Disease Improving Global Outcomes (KDIGO) Clinical Practice Guidelines for Anaemia in Chronic Kidney Disease [8]
- The National Institute for Health and Care Excellence (NICE) guidelines (ng8) [3].

### Background

Anaemia is a common complication of CKD. It is associated with left ventricular dysfunction and heart failure, in addition to a reduction in exercise capacity and quality of life. The use of iron therapies and erythropoiesis stimulating agents (ESAs) has allowed improvement in patients with anaemia of CKD. Newer therapies are under study, but this guideline will not make recommendations on agents such as hypoxia inducible factor stabilisers or hepcidin modulators as data remains preliminary and none of these agents have received a UK marketing authorisation at the time of publication of this guideline.

## Summary of clinical practice guidelines on anaemia of chronic kidney disease

### Evaluating and diagnosing Anaemia in CKD (guidelines 1.1–1.5)

#### Guideline 1.1 – Evaluation of anaemia - screening for anaemia

We suggest that haemoglobin (Hb) levels should be routinely measured to screen for anaemia:

- at least annually in patients with CKD G3 and
- at least twice a year in patients with CKD G4–5 not on dialysis (2B)

#### Guideline 1.2 - evaluation of anaemia – Haemoglobin levels

We recommend that all patients with chronic anaemia associated with chronic kidney disease (CKD) should be investigated for the cause and possible treatment, irrespective of the grade of kidney disease or requirement for renal replacement therapy if:

- their haemoglobin (Hb) levels are less than 110 g/L (less than 105 g/L if younger than 2 years) or
- they develop symptoms attributable to anaemia

This is to ensure the correct diagnosis and management of anaemia. (1A)

#### Guideline 1.3 - evaluation of anaemia - renal function

We suggest that CKD should be considered as a possible cause of anaemia when the glomerular filtration rate (GFR) is <60 ml/min/1.73m^2^. It is more likely to be the cause if the GFR is <30 ml/min/1.73m^2^ (<45/min/1.73m^2^ in patients with diabetes) and no other cause, e.g. blood loss, folic acid or vitamin B_12_ deficiency, is identified. (2B)

#### Guideline 1.4 - evaluation of anaemia - erythropoietin measurement

We recommend that measurement of erythropoietin levels should not routinely be considered for the diagnosis or management of anaemia for patients with CKD. (1A)

#### Guideline 1.5 - Evaluation of anaemia – Baseline investigations

We recommend that initial clinical and laboratory evaluation of anaemia should be performed prior to initiation of treatment for anaemia in CKD patients. (1A)

We recommend that laboratory evaluation should include the following tests (1B):

• Full blood count (FBC) including—in addition to the Hb concentration:

• red blood cell indices:

• mean corpuscular haemoglobin [MCH]

• mean corpuscular volume [MCV]

• mean corpuscular haemoglobin concentration [MCHC])

• white blood cell count and differential count

• platelet count

• Absolute reticulocyte count to assess bone marrow responsiveness (if indicated).

• Test to determine iron status:

• percentage of hypochromic red blood cells (% HRC), but only if processing of blood sample is possible within 6 h **or**

• reticulocyte Hb count (CHr) or equivalent tests e.g. reticulocyte Hb equivalent **or**

• combination of transferrin saturation (TSAT) **and** serum ferritin if the above tests are not available or the person has thalassemia or thalassemia trait

• Serum ferritin to assess iron stores.

• Plasma/serum C-reactive protein (CRP) to assess inflammation.

Based on the initial assessment we recommend in selected cases, the following tests may be useful to diagnose the cause of anaemia (1B):

- Serum B_12_ and serum folate concentrations.
- Tests for haemolysis (plasma/serum levels of haptoglobin, lactate dehydrogenase, bilirubin, Coombs’ test).
- Plasma/serum and/or urine protein electrophoresis.
- Hb electrophoresis.
- Free light chains and bone marrow examination.

### Treatment of Anaemia with iron therapy Anaemia of CKD (guidelines 2.1–2.4)

#### Guideline 2.1 - treatment of Anaemia with iron therapy – Iron repletion

We recommend that patients should be iron replete to achieve and maintain target Hb whether receiving ESAs or not. (1B)

Iron repletion is usually defined as:

- %HRC <6% / CHr >29 pg/ferritin and TSAT (>100 microgram/L and >20%).
- For children, aim for a target ferritin level greater than 100 microgram/L for CKD patients on dialysis as well as CKD patients not on ESA therapy. (ungraded)

#### Guideline 2.2 - treatment of Anaemia with iron therapy - initiation of ESA and iron status

We suggest that ESA therapy should not be initiated in the presence of absolute iron deficiency (ferritin <100 microgram/L) until this is corrected and anaemia persists. In patients with functional iron deficiency iron supplements should be given prior to or when initiating ESA therapy. (2B)

Low serum ferritin is a useful marker to diagnose absolute iron deficiency. Normal or high serum ferritin values (≥100 microgram/L) do **not** exclude iron deficiency, as it could be due to other causes as infection or inflammation.

#### Guideline 2.3 - treatment of Anaemia with iron therapy - route of administration

We suggest that oral iron will, in general, be sufficient to maintain and may be sufficient to attain the Hb within targets in ESA treated CKD patients **not** yet requiring dialysis and in those on peritoneal dialysis (PD). (2B)

For CKD patients **not** requiring haemodialysis, the choice between oral vs. parenteral iron depends on the severity of iron deficiency, the previous response and side effects, the availability of venous access and the need to initiate ESA therapy (2A).

In contrast most haemodialysis patients will require intravenous iron. (2A).

When offering intravenous iron therapy to people **not** receiving in-centre haemodialysis, consider **high dose, low frequency (HD/LF)** IV iron as the treatment of choice for adults and young people when trying to achieve iron repletion, taking into account all of the following:

- the availability of venous access
- preferences of the person with anaemia of CKD or, where appropriate, their family or carers
- nursing and administration costs
- cost of local drug supply
- provision of resuscitation facilities

#### Guideline 2.4 - treatment of Anaemia with iron therapy - upper limit for iron therapy

We recommend that serum ferritin should not exceed 800 microgram/L in patients treated with iron, and to achieve this iron management should be reviewed when the ferritin is >500 microgram/L. (1B)

### Treatment with Erythropoiesis stimulating agents (guidelines 3.1–3.11)

#### Guideline 3.1 - treatment of Anaemia - Erythropoiesis stimulating agents

We recommend that treatment with Erythropoiesis Stimulating Agents (ESAs) should be offered to patients with anaemia of CKD who are likely to benefit in terms of quality of life and physical function and to avoid blood transfusion; especially in patients considered suitable for transplantation. (1B)

#### Guideline 3.2 - treatment of Anaemia - choice of ESA

We recommend that the decision on the choice of ESA is based on local availability of ESAs. (1B)

#### Guideline 3.3 - treatment of Anaemia with ESA therapy - target Hb

We suggest that patients with CKD on ESA therapy should achieve Hb between:

- 100 and 120 g/L in adults, young people and children aged 2 years and older (2B)
- 95 and 115 g/L in children younger than 2 years of age (reflecting the lower normal range in that age

#### Guideline 3.4 - treatment of Anaemia without ESA therapy - target Hb

We suggest that this Hb target range applies exclusively to patients receiving ESA and are not intended to apply to the treatment of iron deficiency in patients receiving iron therapy without the use of ESAs. (2B)

#### Guideline 3.5 - treatment of Anaemia - initial ESA dose

We recommend that the initial ESA dose should be determined by the patient’s Hb level, the target Hb level, the observed rate of increase in Hb level and clinical circumstances. (2B)

#### Guideline 3.6 - treatment of Anaemia with ESA therapy - route of administration

We suggest that the route of ESA administration should be determined by the CKD grade, treatment setting, efficacy, safety, and class of ESA used; subcutaneous (SC) route is the access of choice in non-haemodialysis patients, while convenience may favour intravenous (IV) administration in haemodialysis patients. (2B)

#### Guideline 3.7 - treatment of Anaemia with ESA therapy - frequency of administration

We suggest that the frequency of administration should be determined by the CKD grade, treatment setting and class of ESA. Less frequent administration using long acting ESAs may be the treatment of choice in non– haemodialysis patients. (2B).

#### Guideline 3.8 - treatment of Anaemia with ESA therapy - ESA dose adjustments

We recommend that adjustments to ESA doses should be considered when Hb is <105 or >115 g/L in adults, young people and children aged 2 years and older, in order to balance the benefit and safety to patients given the current evidence base.

These thresholds for intervention should achieve a population distribution centred on a mean of 110 g/L with a range of 100–120 g/L. (2B)

In children younger than 2 years to keep the Hb level within the aspirational range, do not wait until Hb levels are outside the aspirational range before adjusting treatment (for example, take action when Hb levels are within 5 g/L of the range’s limits).

#### Guideline 3.9 - treatment of Anaemia with ESA therapy - ESA dose adjustments

We suggest that ESA doses should ideally be decreased rather than withheld when a downward adjustment of Hb level is desirable (2B).

#### Guideline 3.10 - treatment of Anaemia with ESA therapy

We suggest that ESA administration in ESA-dependent patients should continue during acute illness, surgical procedures or any other cause of hospitalisation, unless there is a clear contra-indication such as accelerated hypertension. (2B)

#### Guideline 3.11 – Caution in prescribing ESA in certain CKD patients sub-group

We suggest exerting extreme caution while prescribing ESA therapy in CKD patients with a history of stroke, or malignancy, particularly in those with active malignancy when cure is the anticipated outcome. (2C)

### Monitoring of therapy (guidelines 4.1–4.7)

#### Guideline 4.1 - monitoring of treatment - Hb during ESA therapy

We suggest that Hb concentration should be monitored every 2–4 weeks in the correction phase and every 1–3 months for stable patients in the maintenance phase.

More frequent monitoring will depend on clinical circumstances. (2B)

#### Guideline 4.2 - monitoring of treatment - iron therapy

We recommend regular monitoring of iron status (every 1–3 months) in patients receiving intravenous iron to avoid toxicity (2B): a serum ferritin consistently greater than 800 microgram/L with no evidence of inflammation (normal CRP) may be suggestive of iron overload. (1B)

#### Guideline 4.3 - monitoring during intravenous iron administration

We recommend that resuscitative medication and personnel trained to evaluate and resuscitate anaphylaxis should be present at each administration of intravenous iron. (1A)

#### Guideline 4.4 - Parenteral iron & infection

We suggest avoiding parenteral iron therapy in patients with active infection (2B).

#### Guideline 4.5 - monitoring of treatment - resistance to ESA therapy

We recommend that inadequate response (‘resistance’) to ESA therapy is defined as failure to reach the target Hb level despite SC epoetin dose >300 IU/kg/week (450 IU/kg/week IV epoetin), or darbepoetin dose >1.5 microgram/kg/week. Hyporesponsive patients who are iron replete should be screened clinically and by investigations for other common causes of anaemia. (1A)

#### Guideline 4.6- evaluation for ESA induced pure red cell Aplasia (PRCA)

- We do not recommend routine screening for anti-erythropoietin antibodies among CKD patients regularly treated with erythropoiesis stimulating agents. (2A)
- We recommend that the diagnosis of ESA induced PRCA should be considered whenever a patient receiving long term ESA therapy (more than 8 weeks) develops all the following (2A):
  • a sudden decrease in Hb concentration at the rate of 5 to 10 g/L per week OR requirement of transfusions at the rate of approximately 1 to 2 per week,
  • normal platelet and white cell counts,
  • absolute reticulocyte count less than 10,000/μl
- We recommend that all ESA therapy should be stopped in patients who develop ESA induced PRCA. (2A)
- We recommend that patients who remain transfusion dependent after withdrawing ESA therapy should be treated with immunosuppressant medications guided by the level of anti EPO antibodies. (2A)

#### Guideline 4.7 - monitoring of treatment - hypertension during ESA therapy

We recommend that blood pressure should be monitored in all patients receiving ESAs and, if present, hypertension be treated by volume removal and/or anti-hypertensive drugs. (1A)

### Anaemia of CKD: Blood transfusion (guidelines 5.1–5.3)

#### Guideline 5.1 - blood transfusion

We recommend that in patients with anaemia of CKD, especially those in whom renal transplantation is an option, red blood cell transfusion should be avoided where possible to minimise the risk of allosensitisation. (1A)

#### Guideline 5.2 - blood transfusion

We recommend if red blood cell transfusion becomes essential (usually in the setting of acute blood loss, acute haemolysis or severe sepsis) transfusions should be based on policies set by local transfusion guidelines rather than Hb targets for ESA therapy in chronic anaemia of CKD. (1B)

#### Guidelines 5.3- blood transfusion

We recommend that renal transplant recipients, or those on the transplant waiting list or patients on immunosuppressive therapy should receive only Hepatitis E negative blood components. (2B)

### Anaemia of CKD: Post transplant Anaemia (guideline 6.1)

We suggest that the treatment guidelines for anaemia in renal transplant patients should be similar to those for CKD patients not on dialysis. (2B)

## Summary of audit measures on anaemia of chronic kidney disease

1. Proportion of CKD patients with eGFR <30 ml/min (using 4 variable MDRD or CKD-EPI) method with an annual Hb level.
2. Proportion of patients starting an ESA without prior measurement of %HRC or CHr (or serum ferritin and TSAT).
3. Proportion of patients on renal replacement therapy with Hb level < 100 g/L who are not prescribed an ESA
4. Each renal unit should audit the type, route and frequency of administration and weekly dose of ESA prescribed
5. The proportion of CKD Grade 4–5 patients with Hb 100–120 g/L
6. The proportion of patients treated with an ESA with Hb > 120 g/L
7. Mean (median) ESA dose in patients maintained on ESA therapy
8. Each renal unit should monitor ESA dose adjustments
9. Proportion of patients with serum ferritin levels <100 microgram/L at start of treatment with ESA
10. Proportion of pre-dialysis and PD patients receiving iron therapy; type: oral vs. parenteral
11. Proportion of HD patients receiving IV iron
12. Prevalence of resistance to ESA among renal replacement therapy patients
13. Proportion of HD patients who received a blood transfusion within the past year

## Rationale for clinical practice guidelines for anaemia of CKD

### Anaemia of CKD (guidelines 1.1–1.6)

#### Guideline 1.1 – Evaluation of anaemia - screening for anaemia

We suggest that haemoglobin (Hb) levels should be routinely measured to screen for anaemia:

- at least annually in patients with CKD G3 and
- at least twice a year in patients with CKD G4–5 not on dialysis (2B)

##### Audit measure

Proportion of CKD patients with eGFR <30 ml/min (using 4 variable MDRD or CKD-EPI) method with an annual Hb level.

##### Rationale

There is insufficient literature to suggest the ideal frequency of Hb testing in CKD patients who are not on ESA therapy. Alternatively data from clinical trials have shown that the rate of Hb decline in these patients is gradual one [9, 10]. In a Canadian study to assess the effect of ESA therapy on left ventricular mass in patients with CKD [10] 172 patients were assigned to either receive therapy with erythropoietin α subcutaneously to maintain or achieve Hb level targets of 120 to 140 g/L, or to the control/delayed treatment group with mean Hb levels of 90 ± 5 g/L. During 2 years follow up a significant proportion of patients eventually required ESA therapy. However, among those who did not require ESA therapy, mean Hb values remained relatively stable throughout the study period. Hb level should be measured at least monthly in CKD G5 haemodialysis patients and every 3 months in CKD G5 peritoneal dialysis patients.

KDIGO 2012 guidelines suggest measurement of Hb at least annually in patients with CKD G3, at least twice per year in patients with CKD G4–5ND and at least every 3 months in patients with CKD G5HD and CKD G5 PD. For those treated with an ESA, they recommend measuring Hb concentration when clinically indicated and at least every 3 months in patients with CKD G3–5ND and CKD G5PD and at least monthly in patients with CKD G5HD

#### Guideline 1.2 - evaluation of anaemia - Haemoglobin level

We recommend that all patients with chronic anaemia associated with chronic kidney disease should be investigated for the cause and possible treatment, irrespective of the grade of kidney disease or requirement for renal replacement therapy if:

- their haemoglobin (Hb) levels are less than 110 g/L (less than 105 g/L if younger than 2 years) or
- they develop symptoms attributable to anaemia

This is to ensure the correct diagnosis and management of anaemia. (1A)

##### Rationale

The Renal Association (RA) and Royal College of Physicians endorse the NICE Guidelines for Chronic Kidney Disease: Managing Anaemia. The reader is referred to these guidelines as well as the European Renal Best Practice (ERBP) for Anaemia in CKD and the KDOQI Guidelines for management of anaemia in CKD. The

KDIGO website (www.kdigo.org) is a useful site of reference for comparison of evidence based guidelines internationally.

Anaemia is defined as having a Hb value below the established cut off defined by the World Health Organisation. Different defined groups have different cut offs. For adults:

- Men and postmenopausal women Hb < 130 g/L
- Premenopausal women Hb < 120 g/L

In 2006, KDOQI modified this definition by giving a single criterion for diagnosing anaemia in adult males (Hb *<*135 g/L, regardless of age) because the decrease in Hb among males aged *>*60 years is often attributable to associated co-morbidities, while KDIGO suggest a diagnosis of anaemia in adults with CKD when the Hb concentration is <130 g/L in males and <120 g/L in females.

Anaemia is defined as a haemoglobin concentration less than the 5th percentile for age. Hb levels vary by age, and many laboratories use adult norms as references; therefore, the patient’s Hb level must be compared with age-based norms to diagnose anaemia [11].

In addition to gender, age and pregnancy other factors influence Hb level including smoking, altitude, race and genetic disorders (thalassemia and sickle cell disease). In CKD a patient’s anaemia should be defined using these same criteria. The degree of renal impairment affects the likelihood of any patient developing anaemia. Although current treatment with ESAs is not recommended unless Hb falls consistently below 110 g/L, other causes of anaemia should be excluded in patients with Hb below normal range. The current definition for anaemia applies to adult patients older than 18 years, of all races and ethnic groups, and living at relatively low altitude (<1000 m or 3000 ft.) [12]. With increasing altitude, endogenous erythropoietin production is increased; as a result, Hb concentration can be expected to increase by about 6 g/L in women and 9 g/L in men for each 1000 m of altitude above sea level [13].

#### Guideline 1.3 - evaluation of anaemia - renal function

We suggest that CKD should be considered as a possible cause of anaemia when the glomerular filtration rate (GFR) is <60 ml/min/1.73m^2^. It is more likely to be the cause if the GFR is <30 ml/min/ 1.73m^2^ (<45 ml/min/ 1.73m^2^ in patients with diabetes) and no other cause, e.g. blood loss, folic acid or vitamin B_12_ deficiency, is identified (2B).

##### Audit measure

Proportion of CKD patients with eGFR <30 ml/min (using 4 variable MDRD or CKD-EPI) with an annual Hb level.

##### Rationale

The prevalence of anaemia in patients with CKD increases as the GFR progressively falls [14]. NHANES III data demonstrate a prevalence of anaemia of 1%, 9% and 33% in CKD patients with an eGFR of 60, 30 and 15 ml/min/1.73m^2^ respectively [14]. UK data of >112,000 unselected patients in the general population showed a population prevalence of CKD G3-G5 of 4.9% [15]. In these patients the prevalence of gender specific anaemia (<120 g/L men: < 110 g/L women) was 12%.

Anaemia is more prevalent among patients with diabetes. In addition, anaemia of CKD develops earlier in patients with diabetes compared with non-diabetics [16–20]. In a cross-sectional study involving over 800 patients with diabetes, anaemia has been found to be two to three times more prevalent in patients with diabetes compared with the general population at all levels of GFR [21].

#### Guideline 1.4 - evaluation of anaemia - erythropoietin measurement

We recommend that measurement of erythropoietin levels should not routinely be considered for the diagnosis or management of anaemia for patients with CKD. (1A)

##### Rationale

In renal anaemia, serum erythropoietin (EPO) levels are lower than appropriate for the degree of anaemia. In CKD patients with anaemia, erythropoietin titres are not lower but may be equal to or even higher than in normal non-anaemic individuals [22–24]. Measurement of erythropoietin level is very rarely helpful.

#### Guideline 1.5 - evaluation of anaemia – Baseline investigations

We recommend that initial clinical and laboratory evaluation of anaemia should be performed prior to initiation of treatment for anaemia in CKD patients. (1A)

We recommend that laboratory evaluation should include the following tests (1B):

• Full blood count (FBC) including—in addition to the Hb concentration:

• red blood cell indices:

• mean corpuscular haemoglobin [MCH]

• mean corpuscular volume [MCV]

• mean corpuscular haemoglobin concentration [MCHC])

• white blood cell count and differential count

• platelet count

• Absolute reticulocyte count to assess bone marrow responsiveness (if indicated).

• Test to determine iron status:

• percentage of hypochromic red blood cells (% HRC), but only if processing of blood sample is possible within 6 h **or**

• reticulocyte Hb count (CHr) or equivalent tests e.g. reticulocyte Hb equivalent **or**

• combination of transferrin saturation (TSAT) and serum ferritin if the above tests are not available or the person has thalassemia or thalassemia trait

• Serum ferritin to assess iron stores.

• Plasma/serum C-reactive protein (CRP) to assess inflammation.

Based on the initial assessment we recommend in selected cases, the following tests may be useful to diagnose the cause of anaemia (1B):

- Serum B_12_ and folate concentrations.
- Tests for haemolysis (plasma/serum levels of haptoglobin, lactate dehydrogenase, bilirubin, Coombs’ test).
- Plasma/serum and/or urine protein electrophoresis.
- free light chains and bone marrow examination.
- Hb electrophoresis.

##### Rationale

Although relative erythropoietin deficiency is common among patients with anaemia and CKD, other potential causes should be identified or excluded. A clinical and laboratory evaluation of the cause of anaemia should precede initiation of ESA therapy.

The recommended laboratory evaluation aims at assessing:

- The degree and cause of anaemia,
- Bone marrow responsiveness, and
- Iron stores and iron availability for erythropoiesis.

Anaemia due to causes other than erythropoietin deficiency should be suspected when:

- The severity of the anaemia is disproportionate to the deficit in renal function,
- There is evidence of iron deficiency,
- There is evidence of haemolysis, or
- There is evidence of bone marrow disorder as manifest by leucopoenia and/or thrombocytopenia.
  - **Assessment of anaemia severity**

In CKD patients not yet requiring dialysis and in those on peritoneal dialysis (PD), the timing of the blood sample draw is not critical because plasma volume in these patients remains relatively constant. In haemodialysis (HD) patients one issue remains to be clarified. Haemoglobin concentrations are routinely measured in dialysis patients before dialysis. This potentially leads to lower haematocrit values as a result of dilution from fluid overload prior to ultrafiltration and an underestimate to actual haemoglobin value.

Interdialytic weight gain contributes to a decrease in Hb level, whereas intradialytic ultrafiltration leads to an increase in Hb level. Thus, a pre-dialysis sample underestimates the euvolaemic Hb level, whereas a post dialysis sample over-estimates the euvolaemic Hb. Indeed changes on haematocrit can vary from the start to the end of dialysis by up to 6% depending of the volume of ultrafiltration. In a study of 68 stable HD patients receiving erythropoietin subcutaneously, average mean pre-dialysis Hb was 10 g/L lower than average post dialysis Hb [25]. There was a strong linear inverse correlation between percentage of change in Hb and haematocrit (Hct) values and percentage of change in body weight. In another study of 49 stable HD patients, among all pre-HD and post-HD Hb values, levels measured at the end of short dialysis intervals were closest to the mean Hb value of the week, derived from calculation of the area under the curve for the readings of the week [26]. In unit based haemodialysis patients receiving thrice weekly dialysis, Hb monitoring performed prior to a mid-week haemodialysis session would minimise Hb variability due to the longer inter-dialytic interval between the last treatment of 1 week and the first of the next.

b.) **Assessment of Bone Marrow Responsiveness**

In general, anaemia of CKD is normochromic and normocytic and is morphologically indistinguishable from the anaemia of chronic illness. Initial assessment of anaemia in CKD patients should aim at identifying other factors that may influence the response to treatment.

In addition to Hb, other indices of the FBC report may provide important clinical information:

- Macrocytosis could be due to folate or vitamin B12 deficiency.
- In addition to anaemia of CKD, microcytosis could be due to iron deficiency or haemoglobinopathies.
- Macrocytosis with leucopoenia or thrombocytopenia could be due to several factors such as alcohol intake, nutritional deficit (vitamin B12 or folate deficiency), or myelodysplasia.
- Serum folate is more prone to variation and can be affected by the patient’s diet immediately prior to blood being taken, alcohol, trauma and other factors therefore occasionally red cell folate may need to be measured where serum folate is equivocal.
- Haemolysis is suggested by the presence of macrocytosis, high lactate dehydrogenase and positive Coombs test.
- The normal absolute reticulocyte count ranges from 40,000 to 50,000 cells/μL. Although it has a significant inter-patient variability, this test may be useful as a semi-quantitative marker of erythropoietic activity.

c.) **Evaluating Iron Status in Anaemic Patients with CKD**

The aim of evaluating iron status is to assess:

1. Iron level in tissue stores and
2. The adequacy of iron utilisation for erythropoiesis.

Serum ferritin level is the only available blood marker of storage iron. There are several tests to assess adequacy of iron for erythropoiesis: TSAT, MCV, MCH, percentage of hypochromic red blood cells (HRC) and reticulocyte Hb content (CHr) or Ret-Hb.

##### Tests limitations

- HRC estimation is a useful test for assessment of iron availability but is limited by the effect of sample storage time and need for special analysers. Long sample storage time (> 6 h) may spuriously increase HRC. Because a fresh sample is needed, this measure may not be practical in routine clinical practice.
- If using percentage of hypochromic red blood cells from a fresh sample is not possible, reticulocyte Hb content (CHr) or Ret-Hb could be a suitable alternative.
- If testing for CHr (or Ret-Hb) is not feasible, it is preferable to test ferritin and TSAT together because the combination provides an important insight into erythropoiesis, iron storage and iron availability to bone marrow.
- Low serum ferritin is diagnostic of iron deficiency. High serum ferritin, in addition to expressing the adequacy of iron stores, could be due to inflammatory conditions. TSAT is influenced by nutritional status, timing and inflammation. TSAT is also limited by high day to day variations.

In patients with CKD not on dialysis, serum ferritin levels less than 25 ng/mL in males and less than 12 ng/mL in females suggest depletion of iron stores as a cause of anaemia; but serum ferritin level is less reliable in the evaluation of iron stores in HD patients, because ferritin level is affected by other factors in addition to iron storage status. In relatively healthy HD patients, before widespread use of IV iron therapy, the finding of a ferritin level less than 50 ng/mL was not uncommon [27] and was associated with absent bone marrow iron in approximately 80% of patients [28]. However, in HD patients with several co-morbidities, absent iron stores may still be found at ferritin levels approaching or even exceeding 200 ng/mL [29].

Iron-deficiency is most likely to contribute to anaemia when TSAT results are less than 20%. However, the clinical utility of TSAT is impaired by the absence of a diagnostic threshold above which deficient iron utilisation can be excluded as a cause of anaemia [30].

There is little information in literature to guide the approach to CKD patients who show laboratory evidence of iron deficiency. Nevertheless, given the high prevalence of GI blood loss due to variety of causes in this patient population, deciding on a subsequent management plan, including endoscopy, depends on the clinical presentation. This supports the recommendation that CKD patients who present with anaemia and iron deficiency should undergo careful clinical assessment prior to the initiation of anaemia therapy [31–33].

Reduced iron availability for erythropoiesis can manifest as low mean corpuscular volume (MCV) and mean corpuscular Hb (MCH), but given the relatively long lifespan of circulating erythrocyte, this test will not reflect the existing availability of iron at the time of testing. Testing the reticulocytes for their Hb content (CHr or Ret-He) may allow more accurate estimation of iron availability, because reticulocytes are present in the circulation for 4–5 days, so give a discrete population to study. Reduced red cell Hb can be reflective of reduced haem availability or globin. Therefore, the red cell analyte values (%HRC, CHr, Ret-He) may be affected by the presence of haemoglobinopathies [30].

### Anaemia of CKD (guidelines 2.1–2.4)

#### Guideline 2.1 - treatment of Anaemia with iron therapy – Iron repletion

We recommend that patients should be iron replete to achieve and maintain target Hb whether receiving ESAs or not. (1B)

Iron repletion in is usually defined as:

- %HRC <6% / CHr >29 pg / ferritin and TSAT (>100 microgram/L and >20%).
- For Children, aim for a target ferritin level greater than 100 microgram/L for CKD patients on dialysis as well as CKD patients not on ESA Therapy. (ungraded)

##### Rationale

A definition of adequate iron status is:

- a serum ferritin
- 200–500 microgram/L in HD patients,
- 100–500 microgram/L in non-HD patients and
- Either <6% hypochromic red cells (HRC), or reticulocyte Hb content >29 pg.
- TSAT > 20%

The aim of iron treatment targets is to optimise anaemia therapy while minimising potential toxicity. Therapy targets aim at:

1. Minimising the ESA dose required to maintain target Hb levels in patients on ESA therapy and;
2. Maximising the Hb level and minimising the need to initiate ESA therapy to achieve target-range Hb levels in patients not on ESA therapy.

Increasing the Hb in anaemic patients places the greatest demand for iron in the erythropoietic tissues. During ESA induction therapy iron requirements will depend on the rate of erythropoiesis, the Hb deficit, and ongoing iron losses. Once the target Hb has been reached and Hb stabilised, the iron requirements will be dependent on ongoing iron losses.

When adequate iron status is achieved, CKD patients on ESA therapy should be given maintenance iron treatment.

- Several studies have reported that the dose of ESA required to achieve and maintain a given Hb level is inversely related to iron stores [34–39]. Iron deficiency (absolute or functional) was the main cause of ESA resistance in the UK but this has now been solved by parenteral iron replacement strategies [40]. The evidence behind the statement that TSAT generally should be maintained at greater than 20% stems from a single RCT comparing higher to lower TSAT targets; patients randomized to a target TSAT of 30% to 50% demonstrated a 40% reduction in ESA dose compared with those assigned to a target of 20% to 30%.
- In a randomised controlled study involving 157 haemodialysis patients comparing iron management based on serum ferritin and transferrin saturation versus CHr, CHr was a markedly more stable analyte than serum ferritin or transferrin saturation. Iron management based on CHr resulted in similar haematocrit and epoetin dosing while significantly reducing IV iron exposure [41].
- In another study involving 164 chronic haemodialysis patients, low CHr (<26 pg) was suggestive of functional iron deficiency. When a subgroup of patients were randomly assigned to receive a single dose of IV iron dextran (1000 mg), A CHr < 26 pg at baseline predicted iron deficiency with a sensitivity of 100% and specificity of 80%. The serum ferritin, transferrin saturation and percentage of hypochromic red blood cells were all less accurate. The time to correction of iron deficiency at the level of the reticulocyte was found to be within 48 h as measured by correction of the mean CHr to >26 pg, and by the shift of the vast majority of the reticulocyte population to CHr > 26 pg within this time span [42].
- In a study comparing TSAT versus CHr as a guide of parenteral iron therapy in 197 Japanese peritoneal dialysis patients, although CHr reflected the iron status more sensitively, TSAT was a better clinical marker for iron supplementation therapy [43]. A cross-sectional study of 72 haemodialysis patients was performed. Mean haemoglobin was 9.6 +/− 0.16 g/dl. Mean haemoglobin content of reticulocytes (CHr) was normally distributed and correlated with MCV, MCH and red cell ferritin. A low CHr identified patients with iron deficiency with normal serum ferritin or transferrin saturation [44].
- Tessitore et al. [45] compared the diagnostic efficiency of different iron markers in chronic haemodialysis patients. Although percentage hypochromia >6% was the best marker to identify responsiveness to intravenous iron; CHr was 78% efficient at cut-off ≤29 pg.
- TSAT and serum ferritin were evaluated in 47 chronic haemodialysis patients with baseline serum ferritin levels <600 ng/ml. Patients were treated with IV dextran (1000 mg over ten haemodialysis treatments). Patients were classified as having iron deficiency if haematocrit value increased by 5% or if their erythropoietin dose decreased by 10% by 2 months. Receiver operator curves demonstrated that none of the iron indices had a high level of utility (both sensitivity and specificity >80%). As such it was concluded that both tests should be interpreted in the context of the patient’s underlying EPO responsiveness. In patients who are responsive to EPO, a transferrin saturation value <18% or serum ferritin level < 100 ng/ml should be used to indicate inadequate iron. When EPO resistance is present, transferrin saturation of <27% or serum ferritin <300 microgram/L should be used to guide iron management [46].
- NICE evaluation of iron therapy in CKD patients suggests that for haemodialysis patients, %HRC > 6 dominated all other iron evaluation strategies (it led both to more QALYs and lower cost). For the other patients, TSAT less than 20% alone or serum ferritin less than 100 micrograms/L alone were the least cost effective strategy, but %HRC was the most cost-effective.
- NICE guidelines on anaemia management in CKD patients suggest to:
  • Use percentage of hypochromic red blood cells (% HRC; > 6%), but only if processing of blood sample is possible within 6 h. Since a fresh blood sample is needed, this test may be difficult to use routinely in clinical practice.
  • If using percentage of hypochromic red blood cells is not possible, use reticulocyte Hb content (CHr; < 29 pg) or equivalent tests – for example, reticulocyte Hb equivalent.
  • If these tests are not available or the person has thalassaemia or thalassaemia trait, use a combination of transferrin saturation (less than 20%) and serum ferritin measurement (less than 100 microgram/L).

We believe that CHr (<29 pg) is more sensitive in determining iron depletion than %HRC. This is because CHr reflects haemoglobin content of young reticulocytes, and therefore reflects iron availability in the preceding few days; while %HRC reflects haemoglobin contents of whole erythrocyte pool, and since senescent erythrocyte tend to get smaller in volume, the test may be affected by the overall rate of erythropoiesis.

- If neither test is available, we recommend testing both serum ferritin and transferrin saturation rather than relying on either test separately [46].
- For Children, a target ferritin level greater than 100 microgram/L for CKD patients on dialysis as well as CKD patients not on ESA Therapy is appropriate [47] (ungraded). There is no evidence that a higher ferritin target of 200 microgram/L is beneficial or safe in paediatric CKD HD patients.

#### Guideline 2.2 - treatment of Anaemia with iron therapy - initiation of ESA and iron status

We suggest that ESA therapy should not be initiated in the presence of absolute iron deficiency (ferritin <100 microgram/L) until this is corrected and anaemia persists. In patients with functional iron deficiency iron supplements should be given prior to or when initiating ESA therapy. (2B)

##### Audit measure

Proportion of patients with serum ferritin levels <100 ng/ml at start of treatment with ESA

##### Rationale

Iron is a required for production of new red cells. Iron must be supplied to the erythropoietic tissue at an adequate rate, particularly if stimulated by ESA therapy. If iron stores are low ESAs can still be used if renal anaemia is a likely contributor to the anaemia as long as iron is made directly available to the erythropoietic tissues coincident with the initiation of ESA therapy.

- For CKD dialysis patients, percentage of hypochromic red blood cells >6%, reticulocyte Hb content <29 pg or are ideal test to assess iron status.
- If these tests are not available or the person has thalassaemia or thalassaemia trait, a combination of transferrin saturation (less than 20%) and serum ferritin measurement (less than 100 microgram/L) could be a suitable alternative

#### Guideline 2.3 - treatment of Anaemia with iron therapy - route of administration

We suggest that oral iron will, in general, be sufficient to maintain and may be sufficient to attain the Hb within targets in ESA treated CKD patients not yet requiring dialysis and in those on peritoneal dialysis (PD). (2B)

For CKD patients not requiring haemodialysis, the choice between oral vs. parenteral iron depends on the severity of iron deficiency, the previous response and side effects, the availability of venous access and the need to initiate ESA therapy (2A).

In contrast most haemodialysis patients will require intravenous iron. (2A).

When offering intravenous iron therapy to people not receiving haemodialysis, consider high dose, low-frequency IV iron as the treatment of choice for adults and young people when trying to achieve iron repletion, taking into account all of the following:

- the availability of venous access
- preferences of the person with anaemia of CKD or, where appropriate, their family or carers
- nursing and administration costs
- cost of local drug supply
- provision of resuscitation facilities

Intravenous iron administered at a low dose and high frequency may be more appropriate for adults who are receiving in-centre haemodialysis.

High dose, low frequency (HD/LF) is considered to be a **maximum** of 2 infusions. For adults this is considered to be a **minimum** of 500 mg of iron in each infusion.

Low dose, high frequency (LD/HF) is considered to be **more than** 2 infusions. For adults, there would typically be 100–200 mg of iron in each infusion.

At the time of publication intravenous iron products available in the UK did not have a UK marketing authorisation for all ages of children and young people for this indication.

Refer to the Summary of Product Characteristics for the prescription of individual iron preparations.

##### Audit measure

- Proportion of pre-dialysis and PD patients receiving iron therapy; type: oral vs. parenteral
- Proportion of pre-D and PD patients who are iron replete
- proportion of HD who are iron replete

##### Rationale

The evidence base for intravenous iron over oral iron in pre-dialysis patients and PD patients is limited. Oral iron, if tolerated, appears to be adequate in most patients particularly in combination with ESA therapy. In patients who appear resistant to ESA therapy on oral iron, or are intolerant of oral iron, a therapeutic trial of IV iron trial seems reasonable.

- One randomised study 188 patients of IV iron (1000 mg iron sucrose in divided doses over 14 days) versus oral iron (ferrous sulphate 325 mg TDS) in pre-dialysis patients demonstrated a greater improvement in Hb outcome in those on IV iron (more patients achieved a Hb increased of >10 g/L) but no difference in the proportion of patients who had to commence ESA after the start of the study [48].
- Two studies in pre-dialysis patients not on ESA (one without oral iron and the other after oral iron therapy) demonstrated improvements in Hb outcome after IV iron [49, 50].
- Oral iron is easy and cheap to prescribe. It seems reasonable to treat patients who have not responded to or been intolerant of oral iron with IV iron.
- Two randomised controlled studies of oral versus IV iron supplementation in pre-dialysis patients receiving concomitant ESAs are in agreement. In the first study of 45 patients with Hb <110 g/L given either ferrous sulphate 200 mg TDS versus 300 mg iron sucrose IV monthly, there was no difference in Hb or ESA dose between the oral and IV group receiving ESA over a mean 5.2 months follow-up [51]. Iron stores were greater in the IV than oral group. Five patients (55%) in the oral iron group had diabetes, compared to none on the IV iron group and this may have confounded the results on iron stores. In addition more patients in the oral iron group were exposed to ACEi/ARBs.
- Similar findings were reported in another study of 96 ND-CKD patients comparing 5 weeks of IV iron sucrose (200 mg every 7 days for a total of 5 doses) versus 29 days of thrice daily oral iron (ferrous sulphate 325 mg TDS). There was no difference in Hb or ESA dose but greater increase in ferritin in the IV group [52].In this study the frequency of gastrointestinal symptoms was greater in the oral iron group than the IV iron group (constipation 34.5% vs. 12.5%; nausea 10.4% vs. 4.2%).
- In PD patients a cross-over study of oral and IV iron demonstrated higher Hb and lower ESA doses with IV iron after 4 months oral [53].
- The relative safety of parenteral iron compared with oral iron was assessed in a study involving patients with stage III and IV CKD and iron deficiency anaemia. Patients were randomly assigned to either oral ferrous sulphate (69 patients to 325 mg three times daily for 8 weeks) or intravenous iron sucrose (67 patients to 200 mg every 2 weeks, total 1 g). The trial was terminated early based on a higher risk of serious adverse events in the intravenous iron treatment group. There were 36 serious cardiovascular events among 19 participants assigned to the oral iron treatment group and 55 events among 17 participants of the intravenous iron group (adjusted incidence rate ratio 2.51 (1.56–4.04)). Infections resulting in hospitalisation had a significantly increased adjusted incidence rate ratio of 2.12 (1.24–3.64). The authors concluded that among non-dialysis patients with CKD and anaemia, intravenous iron therapy could be associated with an increased risk of serious adverse events, including those from cardiovascular causes and infectious diseases [54].
- Conversely; the above finding was not reproduced in another study that involved 626 non dialysis CKD patients with anaemia and iron deficiency not on ESAs. In this study, patients were randomized (1:1:2) to intravenous (IV) ferric carboxymaltose (FCM), targeting a higher (400–600 microgram/L) or lower (100–200 microgram/L) ferritin or oral iron therapy. The primary end point was time to initiation of other anaemia management (ESA, other iron therapy or blood transfusion) or Hb trigger of two consecutive values <100 g/L during Weeks 8–52. The increase in Hb was greater with high-ferritin FCM versus oral iron (*P* = 0.014) and a greater proportion of patients achieved an Hb increase ≥10 g/L with high-ferritin FCM versus oral iron (HR: 2.04; 95% CI: 1.52–2.72; *P* < 0.001). Rates of adverse events and serious adverse events were similar in all groups [55].
- Similarly no safety signal could be detected in another study comparing intravenous iron isomaltoside versus oral iron in stage G5 non dialysis patients. In this study 351 iron-deficient patients were randomized 2:1 to either iron isomaltoside 1000 or iron sulphate administered as 100 mg elemental oral iron twice daily (200 mg daily) for 8 weeks. Haemoglobin response, serum-ferritin and transferrin saturation were significantly increased with IV iron compared with those treated with oral iron. Incidence of adverse drug reactions was not different between both groups. More patients treated with oral iron sulphate withdrew from the study due to adverse events (4.3 versus 0.9%, *P* = 0.2) [56].
- At present oral iron should remain first line treatment among CKD patients not on haemodialysis and IV iron used if patients are intolerant of oral iron or remain absolutely or functionally iron deficient despite oral iron. The further interpretation of these results is limited by several factors including the relative short duration of follow-up and limited data on potential long term adverse effects such as the impact of oxidative stress.
- HD patients have additional iron losses from GI bleeding, blood tests and losses in the dialysis lines that result in iron supplementation requirements that outstrip the capacity of the gut to absorb iron. Maintenance IV iron in HD patients greatly reduces ESA requirements and costs [48, 51, 57–60]. Maintaining iron stores at steady state in a HD population requires 50-60 mg/week of intravenous iron [58]. How this is repleted remains a subject under study. A recent open-label, randomized, multicentre, non-inferiority trial conducted in 351 haemodialysis subjects randomized 2: 1 to either iron isomaltoside 1000 (Group A) or iron sucrose (Group B). Subjects in Group A were equally divided into A1 (500 mg single bolus injection) and A2 (500 mg split dose). Group B were also treated with 500 mg split dose. All treatments showed similar efficacy and safety [61].

#### Guideline 2.4 - treatment of Anaemia with iron therapy - upper limit for iron therapy

We recommend that serum ferritin should not exceed 800 microgram/L in patients treated with iron, and to achieve this iron management should be reviewed when the ferritin is >500 microgram/L. (1B)

##### Rationale

• Iron overload is defined as increased total body iron content with the possible risk of organ dysfunction [62].

• There is no clinically available method that accurately determines total body iron content.

• An elevated serum ferritin does not always correlate with elevations in liver iron content [63, 64].

• Magnetic resonance imaging provides a reliable assessment of tissue iron content in HD patients regularly treated with parenteral iron [65]. However, the clinical relevance of increased liver iron remains unclear.

• Elevated serum ferritin together with elevated serum transferrin saturation remain the most clinically accurate parameter of iron overload in CKD patients.

• Discontinuation of adequate maintenance IV iron when an individual’s ferritin is >500 microgram/L produces a population mean that straddles the 500microgram/L ceiling. Ongoing iron therapy in patients with ferritin >500 microgram/L results in a higher median ferritin outcome.

• Interpretation of iron status results and deciding on the need for further iron therapy should include a concomitant assessment of changes in Hb level and ESA dose over time. Examples:

• A dropping ferritin as well as decreasing Hb levels signifies blood loss e.g. on HD or bowel related anaemia: iron therapy is indicated; further investigation may be required depending on the clinical scenario.

• A decreasing ferritin level after initiation of ESA therapy, with a concomitant rise in Hb level indicates a response to ESA with a shift of iron from stores to bone marrow: further iron therapy is guided by target ferritin level.

• An increasing ferritin level after reduction of ESA dose to bring Hb level down to target range indicates ferritin level is rising as Hb synthesis is dropping: further iron therapy may be postponed.

• A rising ferritin level and a drop in TSAT suggest an inflammatory condition: a source of inflammation may be sought: sepsis, vascular access, surgery, recent hospitalisation: further iron therapy depends on target ferritin level and clinical scenario.

• Ongoing high requirements for IV iron to maintain a given ferritin level also point to ongoing blood loss.

• The finding of a TSAT less than 20% coupled with a ferritin level greater than 500 microgram/L poses a particularly difficult problem for clinicians. This situation may be caused by iron test variability, inflammation, or reticuloendothelial iron blockade. Evidence on the risks and benefits of IV iron therapy in these patients is not well established. The effect of iron therapy in this group of patients was assessed in The Dialysis patients’ Response to IV Iron with Elevated ferritin (DRIVE) trial [66], which evaluated the efficacy of intravenous ferric gluconate in 134 patients with high ferritin (500–1200 microgram/L) and low TSAT levels (≤ 25%) who were anaemic despite a high rHuEPO dose (≥225 IU/kg/week or ≥ 22,500 IU/week). After 6 weeks the patients receiving ferric gluconate (125 mg IV at eight consecutive HD sessions) showed a significant increase in Hb in comparison with controls. However, the study has a number of limitations because, given the small sample size and short follow-up, it provides no information about safety and iron overload.

• Finally it is not known whether treatment of patients with CKD and Hb values >120 g/L in the presence of iron deficiency is beneficial. Ongoing studies such as the Iron and Heart Study (EudraCT number: 2014–004133-16) may provide future data.

### Anaemia of CKD (guidelines 3.1–3.11)

#### Guideline 3.1 - treatment of Anaemia - Erythropoiesis stimulating agents

We recommend that treatment with Erythropoiesis Stimulating Agents (ESAs) should be offered to patients with anaemia of CKD who are likely to benefit in terms of quality of life and physical function and to avoid blood transfusion; especially in patients considered suitable for transplantation. (1B)

##### Audit measure

Proportion of patients on renal replacement therapy (on haemodialysis or peritoneal dialysis for more than 3 months) with Hb level < 100 g/L who are not prescribed an ESA.

##### Rationale

Treatment of anaemia in CKD with ESA can be expensive, takes time to work and carries a small but significant risk to the patient. It is therefore reasonable, as with any therapy, to treat only those who are expected to benefit in the time frame that therapy is being considered. For example, patients with severe sepsis/inflammation/acute bleeding are unlikely to respond.

Patients with a very short life expectancy (days or weeks) are not likely to survive long enough for therapy to provide benefit in terms of an increase in Hb. The clinician and patient should agree on a therapeutic plan and, at an appropriate time, review whether therapy is providing enough benefit to continue treatment.

#### Guideline 3.2 - treatment of Anaemia - choice of ESA

We recommend that the decision on the choice of ESA is based on local availability of ESAs. (1B)

##### Audit measure

Each renal unit should audit the type, route and frequency of administration and weekly dose of ESA prescribed.

##### Rationale

Many studies have been published comparing different ESA products against each other when used at different dosing intervals, by different routes of administration and in different patient groups. All the available products are efficacious when administered according to the manufacturers’ recommendations. The choice of ESA will be dependent upon the clinician and patient agreeing a management plan and local supply arrangements.

#### Guideline 3.3 - treatment of Anaemia with ESA therapy - target Hb

We suggest that patients with CKD on ESA therapy should achieve Hb between:

- 100 and 120 g/L in adults, young people and children aged 2 years and older (2B)
- 95 and 115 g/L in children younger than 2 years of age (reflecting the lower normal range in that age (2B)

#### Guideline 3.4 - treatment of Anaemia without ESA therapy - target Hb

We suggest that these Hb targets apply exclusively to patients receiving ESA and are not intended to apply to the treatment of iron deficiency in patients receiving iron therapy without the use of ESAs. (2B)

##### Audit measures

- The proportion of CKD stage 4–5 patients with Hb 100–120 g/L.
- The proportion of patients treated with an ESA with Hb > 120 g/L.
- Mean (median) ESA dose in patients maintained on ESA therapy.

##### Rationale for guidelines 3.3 and 3.4

- In determining target Hb guidelines it is important to assess potential benefits (in terms of possible improved survival, improvement in health related quality of life (HRQoL) and avoidance of transfusion requirement and hospitalisation) vs. potential harms (increased mortality, increased risk of vascular events).
- Although several studies have shown that higher Hb targets could be associated with improvements in both physical and mental health domains [67], the HRQoL benefits of higher Hb targets diminish over time [67]. In addition, there is no apparent Hb threshold above which there is definitively a quality-of-life improvement in the higher Hb treatment arms.
- Besarab et al. [68] reported a study of normalisation of haemoglobin in 1233 prevalent CKD G5HD patients with high cardiovascular risk on haemodialysis. Normalisation of haemoglobin showed no benefit in risk reduction but did show an improvement in quality of life. The treatment arm showed a trend towards increased risk of death failure (183 deaths and 19 myocardial infarcts, producing 202 primary events, compared to 164 events (150 deaths, 14 myocardial infarcts) and vascular access (39% versus 29%) and the trial was terminated before completion on the grounds that the study was unlikely to show benefit from normalisation.
- Two studies evaluated the effect of ESA on patients not yet on dialysis – CHOIR [69] and CREATE [70]. The outcome of the CHOIR study showed no benefit of higher Hb outcome in CKD patients (GFR 15-50 ml/min) randomised to Hb of 113 g/L vs. 135 g/L. Higher outcome target Hb had an increased risk (using composite end-points of death, myocardial infarction, or hospitalisation for congestive cardiac failure) and no incremental improvement in quality of life [69]. The limitation of this study is that, compared with the group assigned to the lower Hb treatment target, the higher Hb target group showed at baseline a statistically greater proportion of patients with a history of hypertension and coronary artery bypass graft. A report posted by the study sponsor [71] indicates that patients assigned to the higher Hb treatment arm also had a significantly greater severity of congestive heart failure (CHF) at baseline. The results of a multivariate analysis, included in this report, indicate that after adjustment for baseline conditions (CHF by National Health and Nutrition Examination Survey CHF score, atrial fibrillation/flutter, serum albumin level, reticulocyte count, and age), the relationship between treatment assignment and primary composite outcome events is no longer statistically significant (HR, 1.24; 95% confidence interval [CI], 0.95 to 1.62; *p* = 0.11 compared with the unadjusted HR of 1.34; 95% CI, 1.03 to 1.74; *p* = 0.03) [72]. A secondary analysis of the CHOIR trial suggested that higher doses of epoetin α, rather than target Hb per se, were associated with an increased risk of death, myocardial infarction, congestive heart failure or stroke compared with lower epoetin doses, and with poorer outcomes [72]. Another secondary analysis of the CHOIR study found that, among patients with diabetes mellitus, the percentage of patients reaching the primary end point of death, myocardial infarction, congestive heart failure or stroke within 3 years was similar in the high and low haemoglobin arms of the trial (24.8% versus 24.7%, respectively; *p* = 0.249). By contrast, among patients without diabetes mellitus at baseline, 36.4% of patients randomized to the higher Hb target had reached the primary end point after 3 years compared with 24% of those randomized to the lower haemoglobin target (HR 1.70; 95% CI 1.03–2.81; *p* = 0.04). Individuals without diabetes mellitus randomized to the higher haemoglobin target had a significantly greater risk of reaching the primary end point after 3 years than individuals with diabetes mellitus randomized to the lower haemoglobin target [73].
- The CREATE [70] study reported that early correction of anaemia to normal Hb (130-150 g/L vs. 105-115 g/L) did not reduce risk of cardiovascular events. Indeed the hazards ratio for primary endpoints of death from any cause or death from cardiovascular disease consistently (but not significantly) favoured the lower haemoglobin target group. The trend to increase in events appeared to occur after initiation of dialysis but there was no difference in endpoints after censoring of data from patients who started dialysis. Quality of life was significantly better in the higher Hb outcome group. Although GFR was not significantly different between the two groups, more patients started renal replacement therapy earlier in the higher Hb outcome group (*p* = 0.03) with the difference apparent from 18 months. An important limitation of this trial is that the event rate was much lower than predicted; thus, the power to detect a difference in event rates was decreased [70].
- Other important limitation (s) of these trials is that important subgroups of patients enrolled in large trials, such as young adults, patients returning to dialysis after failed renal transplant, or patients with chronic lung disease were not identified or assessed in any of these trials.
- Further analysis of outcome of high target Hb was performed by the KDOQI team [74]. An Evidence Review Team analysed all data from randomized controlled trials of anaemia management in CKD, including, CHOIR, CREATE and other studies. Combining mortality outcomes from eight studies involving 3038 subjects with CKD who were not on dialysis (the CHOIR and CREATE studies contributed most of the weight to the analysis) revealed no difference between the higher and lower Hb target [73], but combining adverse cardiovascular events from six studies involving 2850 subjects showed an increased risk among the patients assigned to the higher Hb targets (a RR of 1.24, 95% CI 1.02–1.51) [74], although it is worth noting that the CHOIR and CREATE studies also contributed most of the weight to the analysis. Among dialysis patients, combining mortality (four studies, 2391 subjects) or cardiovascular outcomes (three studies, 1975 subjects) showed no statistically significant difference between the higher and lower Hb level with The US Normal Haematocrit Study [68] contributing most of the weight to the analysis.
  In the TREAT study [75], 4038 patients with diabetes, chronic kidney disease not on dialysis, and anaemia, were randomly assigned in a 1:1 ratio to darbepoetin α, to achieve Hb level of approximately 130 g/L or to placebo, with rescue darbepoetin α when the haemoglobin level was less than 90 g/L. The primary end points were the composite outcomes of death or a cardiovascular event (nonfatal myocardial infarction, congestive heart failure, stroke, or hospitalization for myocardial ischemia) and of death or end-stage renal disease. After a median follow up of 29 months, there was no difference between the two arms in the primary outcome of death, cardiovascular event or end stage renal disease. Fatal or nonfatal stroke occurred in 101 patients assigned to darbepoetin α and 53 patients assigned to placebo (HR, 1.92; 95% CI, 1.38 to 2.68; *p* < 0.001). The investigators concluded that for many involved in clinical decision making this risk of prescribing an ESA in this patient population will outweigh the potential benefits [75].
- Data from observational studies have, however, not shown increased hazard risk among patients who achieved higher Hb. In one study, data from haemodialysis patients in the UK Renal Registry from 1999 to 2005 were analysed for the relative risk of death at different Hb concentrations. Hb concentrations above the reference range (100–110 g/L) consistently showed a 35% lower relative risk of death, while patients with haemoglobin below 100 g/L had a 28% higher mortality. The greatest mortality was seen in patients with haemoglobin <90 g/L (73% increased risk of death, although due to the small numbers, this was not statistically significant). On the other hand, the lowest death rate was seen in patients with haemoglobin levels between 120 and 139 g/L (64% reduced mortality) [76].
- The effect of cumulative ESA dose was also reported in another retrospective study [77]. In this study, which looked at data from Medicare’s end-stage renal disease program between 1999 and 2007, different US dialysis centres annual anaemia management practice were characterised by estimating their typical use of ESAs and intravenous iron in haemodialysis patients within 4 hematocrit categories. Monthly mortality rates were assessed using Cox proportional hazards regression to correlate centre-level patterns of ESA and iron use with 1-year mortality risk in 269,717 incident haemodialysis patients. Monthly mortality rates were highest in patients with haematocrit less than 30% (mortality, 2.1%) and lowest for those with haematocrit of 36% or higher (mortality, 0.7%). After adjustment for baseline case-mix differences, dialysis centres that used larger ESA doses in patients with haematocrit less than 30% had lower mortality rates than centres that used smaller doses (highest vs. lowest dose group: HR, 0.94; 95% CI, 0.90–0.97). Centres that administered iron more frequently to patients with haematocrit less than 33% also had lower mortality rates (highest vs. lowest quintile, HR, 0.95; 95% CI, 0.91–0.98). However, centres that used larger ESA doses in patients with haematocrit between 33% and 35.9% had higher mortality rates (highest vs. lowest quintile, HR, 1.07; 95% CI, 1.03–1.12). More intensive use of both ESAs and iron was associated with increased mortality risk in patients with haematocrit of 36% or higher [77].
- The findings of all the above studies have obviously made it difficult to define a safe target Hb in CKD patients treated with ESA. As a result Target Hb in this patient group has been the subject of extensive debate in the literature:
  • KDIGO suggest that for adult CKD patients on dialysis, ESA therapy could be used to avoid having the Hb concentration fall below 90 g/l by starting ESA therapy when the haemoglobin is between 90 and 100 g/L.
  • The Anaemia Working Group of ERBP expressed its view that Hb values of 110–120 g/L should be generally sought in the CKD population without intentionally exceeding 130 g/L In low-risk patients (i.e. in younger patients with very few comorbidities). In those with ischaemic heart disease with worsening ischaemic symptoms associated with anaemia, or in those in whom a clear benefit on quality of life can be foreseen, the start of ESA therapy could be considered at higher Hb values but not exceeding 120 g/L. In high-risk patients, including those with asymptomatic ischaemic heart disease, treatment initiation with ESA should be started at Hb values between 90 and 100 g/ L in order to maintain a Hb value ∼100 g/L during maintenance therapy [78].
  • NICE guidelines on managing anaemia in CKD patients suggest maintaining the “aspirational” Hb range between 100 and 120 g/L for adults. The rationale behind choosing a wide target Hb range (100–120 g/L) for this guideline is that when the target Hb level is narrow (i.e.10 g/L), variability in achieved Hb levels around the target is high, the fraction of prevalent patients with achieved Hb levels within the target range is low and ESA dose titration is required frequently during maintenance therapy.
  • The health economics of anaemia therapy using ESAs has been subject to a NICE systematic review which concludes that treating to a target Hb 100-120 g/L is cost effective in HD patients. Table 1 summarises the mean Hb data for prevalent UK dialysis patients from the Thirteenth (2010) and Seventeenth (2013) UK Renal Registry Reports.

**Table 1:** Hb data for UK prevalent HD patients [79]

	Median Hb	Hb > 100 g/L	Hb 100–120 g/L	Interquartile Hb range	Hb >110 g/L and not on ESA
2010	115 g/L	85%	53%	105–123 g/L	10%
2013	112 g/L	83%	59%	103–120 g/L	11%
  • The Medicines and Healthcare products Regulatory Agency (MHRA) guidance (2007) notes that using ESAs to achieve Hb levels greater than 120 g/L is associated with an increased risk of death and serious cardiovascular events in people with CKD. The MHRA advises that Hb levels greater than this should be avoided, and that patients should be monitored closely to ensure that the lowest approved dose of ESA is used to provide adequate control of the symptoms of anaemia. Use of ESAs to achieve Hb levels greater than 120 g/L is not consistent with UK marketing authorisations for ESAs. Informed consent should be obtained and documented [80].

#### Guideline 3.5 - treatment of Anaemia - initial ESA dose

We recommend that the initial ESA dose should be determined by the patient’s Hb level, the target Hb level, the observed rate of increase in Hb level and clinical circumstances. (2B)

##### Rationale

For initiation of ESA therapy, several points are to be considered:

- Type(s) of licensed ESAs available
- Initial ESA dose
- ESA dose adjustment: dose required for Hb correction vs. maintenance
- Route of ESA administration
- Frequency of ESA administration that best fit patient requirements and achieve maximal convenience
- Patient monitoring for the anticipated response in terms of Hb rise, rate of Hb rise, possible adverse effect (e.g. hypertension).

In general, the aim of initial ESA therapy is to achieve a rate of increase in Hb levels of 10 to 20 g/L per month. This rate of rise is considered safe as evidenced from interventional trials on ESA naïve patients [81–83]. In CKD patients with initial Hb levels less than target range, these trials have shown the mean initial rate of Hb level increase to be in the range of 7 to 25 g/L in the first 4 weeks. This rate of Hb increase is affected by the patient population, iron status, initial ESA dose, and the frequency and route of ESA administration.

#### Guideline 3.6 - treatment of Anaemia with ESA therapy - route of administration

We suggest that the route of ESA administration should be determined by the CKD grade, treatment setting, efficacy, safety, and class of ESA used; subcutaneous (SC) route is the access of choice in non-haemodialysis patients, while convenience may favour intravenous (IV) administration in haemodialysis patients (2B).

##### Audit measure

Each renal unit should audit the type, route and frequency of administration and weekly dose of ESA prescribed.

##### Rationale

In the outpatient setting, SC administration is the only routinely feasible route of administration for non HD CKD patients. For HD patients, either SC or IV administration is feasible. Among short-acting ESAs, subcutaneous administration is associated with approximately 30% reduction in dose requirements compared to that of IV administration for the same target Hb outcome. This has been proven in a large multi-centre RCT on long term HD patients who had their haematocrit maintained within target range while on epoetin α either via SC or IV route. Patients were then randomised to IV or SC route. Upon randomization, ESA doses were first decreased to allow haematocrit levels to decrease to less than target range. Doses were titrated upward to again achieve target haematocrit levels, and then were adjusted to maintain haematocrit in the target range during a 26-week maintenance phase. Among 107 patients who completed the trial, those assigned to SC route showed 27% lower ESA doses than those assigned to IV administration [84]. However, not all patients showed a dose decrease after conversion from IV to SC, and some patients showed a dose increase.

Among long-acting agents, efficacy of SC administration appears to be equivalent to that of IV route at the examined dosing frequencies [85–88].

#### Guideline 3.7 - treatment of Anaemia with ESA therapy - frequency of administration

We suggest that the route of ESA administration should be determined by the CKD grade, treatment setting, efficacy, safety, and class of ESA used; subcutaneous (SC) route is the access of choice in non-haemodialysis patients, while convenience may favour intravenous (IV) administration in haemodialysis patients (2B).

##### Rationale

The frequency of ESA administration should be determined by the CKD treatment setting and the class of ESA. Maximum efficacy is achieved by using the dosing intervals that are ESA class specific. In HD patients receiving SC short-acting ESA therapy, ESA efficacy is maximal when the drug is given thrice weekly. ESA efficacy decreases and dose requirement increases when the dosing frequency is extended from thrice-weekly to once-weekly administration [89]. Increasing the time interval between dosages of long acting ESAs could also result in an increase in dose requirements [90].

#### Guideline 3.8 - treatment of Anaemia with ESA therapy - ESA dose adjustments

We recommend that adjustments to ESA doses should be considered when Hb is <105 or >115 g/L in adults, young people and children aged 2 years and older, in order to balance the benefit and safety to patients given the current evidence base.

These thresholds for intervention should achieve a population distribution centred on a mean of 110 g/L with a range of 100–120 g/L (2B).

In children younger than 2 years, adjust ESA dose before Hb level is outside the target range to ensure Hb level is maintained within that range (ungraded).

#### Guideline 3.9 - treatment of Anaemia with ESA therapy - ESA dose adjustments

We suggest that ESA doses should ideally be decreased rather than withheld when a downward adjustment of Hb level is required (2B).

#### Guideline 3.10 - treatment of Anaemia with ESA therapy

We suggest that ESA administration in ESA-dependent patients should continue during acute illness, surgical procedures or any other cause of hospitalisation, unless there is a clear contra-indication such as accelerated hypertension (2B).

##### Rationale for guidelines 3.8–3.10

- The NICE Guidelines for anaemia management in chronic kidney disease recommend an “aspirational” Hb of 100–120 g/L. It is anticipated that if a population Hb distribution is centred on this outcome with a mean of 110 g/L, then 85% of the population will have Hb > 100 g/L.
- In HD patients, withholding ESA doses for Hb levels greater than the target range is associated with subsequent downward Hb excursions often to levels less than target Range [91]. The time between withholding ESA doses and return of Hb to target range is variable and unpredictable. In HD patients with Hb values greater than 140 g/L, the median time for Hb to return to 120 g/L or less after withholding of a SC-administered ESA is 7–9 weeks. The difference between withholding long and short acting ESAs on the rate of Hb reduction is not significant.
- ESA dose adjustment may be higher during initiation (or titration after switch between different ESAs) than maintenance phases of ESA therapy. In a randomized double blind trial comparing a short-acting ESA with a long-acting ESA in haemodialysis patients previously receiving epoetin α, dose adjustments were made in 25% increments or decrements of the baseline dose, aiming to maintain individual Hb concentrations within a range of 90 to 130 g/L [92]. Approximately 70% of patients required dose adjustment in the 20-week titration period, and 50% required dose adjustment during the 8 week maintenance period.

#### Guideline 3.11 – Caution in prescribing ESA in certain CKD patients sub-group

We suggest exerting extreme caution while prescribing ESA therapy in CKD patients with a history of stroke, or malignancy, particularly in those with active malignancy when cure is the anticipated outcome (2C).

##### Rationale

- In the TREAT study, there was an increased risk of stroke in the high ESA group (HR 1.92; 95% CI 1.38–2.68): 5.0% of the high Hb group had a stroke compared to 2.6% in the placebo group (*P* < 0.001). Venous thrombo-embolic events occurred significantly more frequently in the high Hb arm (2.0%) compared to the placebo arm (1.1%, *P* = 0.02).
- A post-hoc analysis of TREAT study showed that: 7.4% of those with a history of malignancy at baseline died from cancer in the ESA arm compared to 0.6% in the placebo arm (*P* = 0.002) [93].

In a meta-analysis comparing possible adverse events related to ESA therapy, The higher Hb concentrations in ESA treated CKD patients increased risk for stroke (RR 1.51, 95% CI 1.03–2.21), hypertension (RR 1.67, 95% CI 1.31–2.12), and vascular access thrombosis (RR 1.33; 95% CI 1.16–1.53), and possibly the risk of death (RR 1.09; 95% CI 0.99–1.20), serious cardiovascular events (RR 1.15, 95% CI 0.98–1.33) or ESRD (RR 1.08; 95% CI 0.97–1.20) [94]. However the risk of stroke was independent of Hb level or dose of ESA suggesting other factors such as iron deficiency [95].

- Patients with neoplasia who received ESA in randomised clinical trials had an increased risk of tumour progression and reduced overall survival compared with study controls [96].
- The MHRA advised that r-HuEPOs should not be given to patients with cancer who do not fulfil the criteria in the authorised cancer indications, and that patients should be monitored closely to ensure that the lowest approved dose of r-HuEPO is used to adequately control of symptoms of anaemia [96].
- The joint guideline from the American Society of Clinical Oncology and the American Society of Haematology [97] recommend using ESA therapy with great caution in patients with active malignancy, particularly when cure is the anticipated outcome.
- NICE evaluated the efficacy and safety of ESA in treating anaemia in cancer patients receiving chemotherapy [98]. Although NICE researchers identified 23 randomised controlled trials evaluating the effectiveness and safety of erythropoiesis-stimulating agents (ESAs) for treating cancer treatment-related anaemia. NICE assessment focused only on trials that evaluated ESAs at a starting dose reflecting the current licence (Hb <100 g/L). In total 16 studies were included in the analysis of the outcome related to anaemia and 7 trials in the outcome related to overall survival. NICE analysis of available trials concluded that erythropoiesis-stimulating agents are recommended, within their marketing authorisations, as options for treating anaemia in people with cancer who are having chemotherapy. ESAs were effective in increasing haemoglobin concentrations, improving haematological responses, reducing the need for blood transfusions and improving health-related quality of life, but that it could not assume that ESA treatment either prolonged or shortened survival compared with treatment without an ESA [98].

### Anaemia of CKD (guidelines 4.1–4.5)

#### Guideline 4.1 - monitoring of treatment - Hb during ESA therapy

We suggest that Hb concentration should be monitored every 2–4 weeks in the correction phase and every 1–3 months for stable patients in the maintenance phase.

More frequent monitoring will depend on clinical circumstances. (2B)

##### Rationale

It is important to closely monitor Hb response to treatment to monitor for possible adverse events and plan ESA dose modification. More frequent Hb monitoring may be needed for patients with unstable Hb, out of target Hb level, anticipated Hb drop due to blood loss/haemolysis, infection or suboptimal dialysis.

The response to ESA therapy varies widely between different patient groups and individuals within those groups. In addition, an individual’s response can vary greatly dependent on other clinical variables. During ESA initiation therapy, after drug dose adjustments or changes in an individual’s clinical condition, more frequent monitoring is advised in order that under-treatment (ongoing anaemia) and overtreatment (rapidly rising Hb/hypertension or polycythaemia) may be avoided [99].

#### Guideline 4.2 - monitoring of treatment - iron therapy

We recommend regular monitoring of iron status (every 1–3 months) in patients receiving intravenous iron to avoid toxicity (2B): a serum ferritin consistently greater than 800 microgram/L with no evidence of inflammation (normal CRP) may be suggestive of iron overload (1B).

##### Rationale

Intravenous iron therapy in particular has potential risks as well as benefits. Toxicity associated with high ferritin outcomes was originally reported in the context of multiple transfusions in the pre-ESA era. The risk persists that intravenous iron may reproduce similar toxicity and thus regular monitoring during therapy is required. Similarly with ongoing iron losses on HD regular monitoring to avoid worsening iron deficiency is required. The safety of persistently very high ferritin levels remains unknown. In a cohort of 58,058 prevalent haemodialysis patients in the USA, both all-cause and cardiovascular mortality had increasing rates across increasing ferritin levels, whereas the opposite (inverse) association was observed for TSAT increments. Serum ferritin levels between 200 and 1200 microgram/L and iron saturation ratio between 30 and 50% were associated with the lowest all-cause and cardiovascular death risks. However, association studies are biased by the fact that serum ferritin is also a marker of inflammation. In unadjusted, time-varying model, serum ferritin >800 microgram/L during each quarter was associated with increased death rate [100]. Significant iron overload in the liver and spleen (assessed through *T*_2_ magnetic resonance) has been described in 19 of 21 HD patients with serum ferritin >1000 microgram/L and severe comorbidities who were treated with IV iron. Similarly, Rostoker et al. prospectively studied a cohort of 119 fit HD patients who were receiving iron and ESA therapy and measured their liver iron content by means of *T*_1_ and *T*_2_ magnetic resonance. Mild to severe hepatic iron overload was observed in 84% of the patients, 36% of whom had severe iron overload approaching that found in haemocromatosis [101].

Clinical settings in which more frequent iron testing may be necessary include the following:

1. Initiation of ESA therapy
2. Achieving less-than-target Hb level during ongoing ESA therapy
3. Recent bleeding
4. After surgery
5. After hospitalization
6. Monitoring response after a course of IV iron
7. Evaluation for ESA hypo-responsiveness

#### Guideline 4.3 - monitoring during intravenous iron administration

We recommend that resuscitative medication and personnel trained to evaluate and resuscitate anaphylaxis should be present at each administration of intravenous iron. (1A)

##### Rationale

• All forms of IV iron may be associated with acute adverse events (AEs).

• Immune mechanisms (including IgE-mediated responses or complement activation-related pseudo-allergy) may have a role in some cases [102].

• Anaphylactoid reactions appear to occur more frequently with high molecular weight iron dextran [103].

• Labile or free iron reactions occur more frequently with non-dextran forms of iron [104].

• The rate of life-threatening reactions to iron dextran administration is 0.6% to 0.7% [105, 106].

• In one study, a total of 2534 haemodialysis patients were directly observed after double-blind exposure to intravenous sodium ferric gluconate (SFGC) or placebo. One patient in each of the SFGC and placebo groups experienced anaphylactoid reactions. Additional cases with characteristics possibly consistent with anaphylaxis occurred in 0.4% of intravenous SFGC–treated patients and 0.1% of placebo-treated patients. The results suggest that there is a relatively low rate of anaphylaxis with non-dextran irons and that the reactions are generally easily managed [107].

• The MHRA has issued an updated guidance on the use of parenteral iron. This was in response to concerns raised as a result of serious and rarely fatal hypersensitivity reaction, particularly in pregnant women. These reactions can occur even when a previous administration has been tolerated (including a negative test dose). The risk of hypersensitivity is increased in patients with: known allergies (including drug allergies); immune or inflammatory conditions (e.g., systemic lupus erythematous, rheumatoid arthritis); or those with a history of severe asthma, eczema, or other atopic allergy. As a result the MHRA updated guidelines recommend that [108]:

• IV iron should be administered in strict accordance with the posology and method of administration described in the product information for each individual product (note that advice varies between products).

• Caution is needed with every dose of intravenous iron that is given, even if previous administrations have been well tolerated.

• IV iron products should only be administered when staff trained to evaluate and manage anaphylactic or anaphylactoid reactions—as well as resuscitation facilities—are immediately available.

• Patients should be closely monitored for signs of hypersensitivity during and for at least 30 min after every administration of an IV iron product.

• In patients with increased risk of hypersensitivity, treatment with IV iron products should only be considered if the benefits are clearly judged to outweigh the potential risks.

#### Guideline 4.4- Parenteral iron & infection

We recommend avoiding parenteral iron therapy in patients with active infection (2B)

##### Rationale

- Parenteral iron administration to haemodialysis patients has been shown to result in a reduction of circulating TNFα levels [109]. In addition, chronic iron loading has been associated with an impaired immune response of circulating monocytes to ex vivo stimulation with LPS [110]. Excess iron inhibits anti-microbial effector pathways of macrophages [110, 111]. This is exerted via blockade of LPS and interferon-gamma (IFNJ) inducible immune pathways, while production of macrophage de-activating cytokines such as interleukin-10 (IL10) is increased [112, 113]. The effect of iron on immune function could be dependent on the iron preparation; one study have shown that iron sucrose had more prominent effects on monocyte differentiation than other clinically available compounds [114].
- Ishida and Johansen critically reviewed available literature regarding the association between iron and infection in HD patients [115]. The authors identified studies that evaluated the association between the risk of infection, serum ferritin levels (13 studies) and iron usage (24 studies). Thirteen studies with sample sizes ranging from 61 to 2662 have examined the link between serum ferritin and infection in haemodialysis patients. Among the 13 studies, nine studies reported an association and four studies did not find an association between serum ferritin and infection. Among the studies that identified an association, high serum ferritin (typically defined as >500 or 1000 microgram/L) was associated with higher incidence of bacterial infection or infection-related mortality. The incidence of bacterial infection ranged from 0.34 to 0.59 infections per patient-year (in studies evaluating the rate of infection) and 0.93% to 61.9% (in studies evaluating the proportion with infection) in the higher serum ferritin groups and 0.09 to 0.18 infections per patient-year and 0% to 37% in the lower serum ferritin groups. The authors concluded that these studies suggest an excess of 16 to 50 infections per 100 patient-years in the higher compared with the lower serum ferritin groups. In studies that expressed the association between serum ferritin and bacterial infection as ratios, higher serum ferritin was independently associated with a 1.5 to 3.1-fold higher incidence of bacterial infection or infection-related mortality. Among the 24 studies that evaluated the relationship between iron therapy and infection, 22 studies were observational with sample sizes ranging from 21 to 309,219 patients. Twelve of these studies found an association between any iron usage, higher dose or frequency of iron usage and infection or infection-related mortality.
- One study compared mortality with different dosing patterns of IV iron [116]. Based on data from 117,050 HD patients, the study evaluated the effect of bolus versus maintenance IV iron dosing during repeated 1-month exposure periods on risks of mortality and infection-related hospitalization during the subsequent 3 months. In multivariable additive risk models, compared to maintenance dosing (median monthly dose 200 mg), bolus dosing (median 700 mg) was associated with an increased risk of infection-related hospitalization (risk difference, 25 additional events/1000 patient-years; 95% CI, 16 to 33), with the risk being largest among patients with a catheter or history of recent infection. An association between bolus dosing and infection-related mortality was also observed. In contrast, maintenance and low-dose iron (125 mg) dosing were not associated with increased risks of infection-related hospitalization or mortality outcomes when compared with no iron.
- A multicentre study prospectively evaluated the association between serum ferritin levels and IV iron usage with adverse outcomes and mortality among 1086 Japanese chronic HD patients. By using Cox proportional hazard models and time-dependent variables, there was a significantly higher risk of infection with higher (above 100 microgram/L) compared to lower (below 100 ng/dl) serum ferritin levels, and with high (≥50 mg/week) and even low (<50 mg/week) doses of IV iron compared with no IV iron; they also reported significantly higher risk of death among patients with high-amplitude ferritin fluctuations (serum ferritin level consistently above 100 microgram/L or upward trend from below to above 100 microgram/L) compared with those with low ferritin level [117].
- In a study involving 626 patients with pre-dialysis CKD patients. Patients were treated with intravenous ferric carboxymaltose (with a high and low ferritin target) or oral iron for 52 weeks. The percentage of deaths, myocardial infarctions, and infections was not significantly different between oral iron–treated and IVI-treated patients. However, the study was not powered to evaluate safety of parenteral iron.
- In a study evaluating the safety of parenteral iron therapy in10,169 haemodialysis patients in the United States; after adjusting for 23 demographic and comorbidity characteristics among 5833 patients included in the multivariable analysis; bills for ≤10 vials of iron over 6 months showed no adverse effect on survival when compared with none, but bills for >10 vials showed a statistically significant elevated rate of death. Bills for ≤10 vials of iron over 6 months also showed no significant association with hospitalization (adjusted = 0.92; 95% CI, 0.83 to 1.03; *P* = 0.15), but bills for >10 vials showed statistically significant elevated risk. More intensive dosing was associated with diminished survival and higher rates of hospitalization, even after extensive adjustment for baseline comorbidity. [118]
- A subsequent analysis of 32,566 Fresenius Inc. haemodialysis patients by the same authors did not confirm an association between IVI dose and risk of death after adjusting for time-varying measures of iron treatment and fixed and time-varying measures of morbidity [119]
- Kalantar-Zadeh et al. studied 58,058 DaVita Inc. dialysis patients. For patients who received 400 mg of IVI per month, the risk for death was found to be lower compared with patients with no IVI administered. By contrast, doses >400 mg per month were associated with increased risks of death [120].
- Kshirsagar et al. studied 117,050 haemodialysis patients. No association was found between dose of IVI and short-term risk of myocardial infarction, stroke, or death [121].
- A prospective observational study by Hoen et al. followed 988 haemodialysis patients from 19 French centres for 6 months. There were 51 episodes of bacteraemia, but no association with either IVI dosing or serum ferritin concentration was detected [122]A more recent study from the same group in 985 dialysis patients, demonstrated no increase in infection rates [123].

#### Guideline 4.5 - monitoring of treatment - resistance to ESA therapy

We recommend that inadequate response (‘resistance’) to ESA therapy is defined as failure to reach the target Hb level despite SC epoetin dose >300 IU/kg/week (450 IU/kg/week IV epoetin), or darbepoetin dose >1.5 microgram/kg/week. Hyporesponsive patients who are iron replete should be screened clinically and by investigations for other common causes of anaemia. (1A)

##### Audit measure

Prevalence of resistance to ESA among renal replacement therapy patients.

##### Rationale

Extensive publications are available on the topic of resistance to ESA therapy including the Revised European Best Practice Guidelines [124] which defines ESA resistance as above. Failure to respond at an earlier stage in therapy should however raise suspicion of ESA resistance.

Comparison of the individual Hb outcome achieved and the dose of ESA used can provide a useful way of highlighting individuals that are ESA resistant during local unit audit [125, 126]. ESA therapy is efficacious in most patients. However many conditions and treatment variables can cause or explain apparent resistance to ESA therapy. Adequate investigation and management of these underlying conditions is crucial in achieving satisfactory outcome haemoglobin values as well as requiring therapy in their own right.

#### Guideline 4.6- evaluation for ESA induced pure red cell Aplasia (PRCA)

• We do not recommend routine screening for anti-erythropoietin antibodies among CKD patients regularly treated with erythropoiesis stimulating agents. (2A)

• We recommend that the diagnosis of ESA induced PRCA should be considered whenever a patient receiving long term ESA therapy (more than 8 weeks) develops all the following (2A):

• a sudden decrease in Hb concentration at the rate of 5 to 10 g/L per week OR requirement of transfusions at the rate of approximately 1 to 2 per week,

• normal platelet and white cell counts,

• absolute reticulocyte count less than 10,000/μl

• We recommend that all ESA therapy should be stopped in patients who develop ESA induced PRCA. (2A)

• We recommend that patients who remain transfusion dependent after withdrawing ESA therapy should be treated with immunosuppressant medications guided by the level of anti EPO antibodies. (2A)

##### Rationale

Anti-erythropoietin antibody associated pure red cell aplasia (PRCA) is a very rare cause of resistance characterised by transfusion dependency, low reticulocyte count (<1%), lack of proerythroid progenitor cells in the bone marrow and neutralising anti-erythropoietin antibodies [127]. ESA induced PRCA is a very rare condition, with the overall incidence of reported cases between 1989 and June 2004 was 1.6 per 10,000 patient-years of subcutaneous exposure [128], and 0.02 per 10,000 patient-years of intravenous exposure [129]. Nevertheless, most reported cases of anti-erythropoietin antibody-associated PRCA have occurred in CKD patients who have received the drug subcutaneously [130–132].

Pure red cell aplasia (PRCA) due to anti-erythropoietin (EPO) antibodies should be suspected in an individual who has previously responded to EPO if the haemoglobin (Hb) level declines by >20 g/l per month or the reticulocyte count is <20,000/uL [131].

PRCA is specifically characterized by the following clinical features [132]:

• A drop in Hb level of >7 to 10 g/L per week without transfusions or transfusion requirement of at least one unit per week to maintain adequate Hb, despite continued use of ESA at high doses.

• Markedly reduced reticulocyte count (<10,000/uL).

• Normal platelet and white blood cell count.

• Elevated serum transferrin saturation and serum ferritin.

• Rarely, allergic urticarial skin reactions at sites of earlier subcutaneous EPO injections have been described [133].

• The diagnosis of PRCA is established by:

• Bone marrow examination: which confirms severe hypoplasia of erythroid precursors (<5%).

• The presence of anti-erythropoietin antibodies:

• There are several available tests to detect antibodies to erythropoietin, with varying sensitivities and specificities [134].

• Patients with suspected ESA induced PRCA who test positive using binding antibodies should have the diagnosis confirmed with the definitive testing for neutralizing antibodies [135].

ESA induced PRCA is an immune mediated process. While spontaneous remissions after cessation of EPO therapy have been reported, immunosuppressive therapy is usually needed in most cases [136]. One study evaluated 170 CKD patients who developed epoetin-associated PRCA [137]. Of the 34 patients who received epoetin after the onset of PRCA, 56% recovered epoetin responsiveness; the highest rate of epoetin responsiveness was observed among those who had no detectable anti-erythropoietin antibodies at the time of epoetin administration (89%). The study also reported that the highest recovery rates were among those treated with immunosuppressive therapy, particularly a combination of cyclophosphamide and prednisone [137]. Other options such as rituximab, danazol or even plasma exchange may be considered.

Verhelst et al. [138] compared various immunosuppressive agents in 37 patients with antibody mediated PRCA compared to 10 with no treatment and found benefit with cyclophosphamide, plasma exchange and ciclosporin and also transplantation.

Given these data, it is advisable that retreatment with ESA may be considered in patients with a history of PRCA only if anti-EPO antibody level is no longer detectable. In addition, if epoetin therapy is to be reconsidered for these patients, only the intravenous rather than the subcutaneous route should be considered for drug administration.

ESA induced PRCA is now part of RaDaR, the rare disease registry [139].

#### Guideline 4.7 - monitoring of treatment - hypertension during ESA therapy

We recommend that blood pressure should be monitored in all patients receiving ESAs and, if present, hypertension be treated by volume removal and/or hypotensive drugs. (1A)

##### Rationale

Hypertension is the most common complication in CKD and can be aggravated by ESA treatment. Early studies demonstrated higher incidence rates of hypertension though ESA doses used were higher and Hb responses faster in these trials. It is now more common to start at low doses and increase gradually according to response. The commonest cause of hypertension in CKD is not ESA therapy. Exacerbation of hypertension in ESA therapy patients may be associated with polycythaemia or rapidly rising haemoglobin levels. These complications should be looked for in hypertensive patients but in the absence of these complicating factors and in the absence of severe hypertension, ESA therapy can usually continue. Hypertension should be adequately controlled prior to initiating ESA therapy. ESA therapy should be discontinued in malignant hypertension.

### Anaemia of CKD (guidelines 5.1–5.3)

#### Guideline 5.1 - blood transfusion

We recommend that in patients with anaemia of CKD, especially those in whom renal transplantation is an option, red blood cell transfusion should be avoided if possible to minimise the risk of allosensitisation (1A).

#### Guideline 5.2 - blood transfusion

We recommend if red blood cell transfusion becomes essential (usually in the setting of acute blood loss, acute haemolysis or severe sepsis) transfusion should be based on policies set by local transfusion guidelines rather than Hb targets for ESA therapy in chronic anaemia of CKD (1B).

#### Guidelines 5.3- blood transfusion

We recommend that renal transplant recipients, those on the transplant waiting list or patients on immunosuppressive therapy should receive only Hepatitis E negative blood components (2B).

##### Audit measure

Proportion of HD patients who received a blood transfusion within the past year.

##### Rationale for guidelines 5.1–5.3

CKD results in chronic anaemia, the degree of which usually reflects the severity of CKD. As with any chronic anaemia, patients tend to deal with this by various compensatory mechanisms. Blood transfusion is rarely an acute requirement except in emergencies such as acute blood loss, acute haemolysis or severe sepsis/inflammation. Hence the risk benefit ratio of the intervention needs to be analysed before prescribing a red blood cell transfusion to treat anaemia in patients with chronic kidney disease.

The potential risks associated with blood transfusion include transfusion reactions, iron overload and transfusion acquired infections. In the presence of severe chronic anaemia, transfusion may lead to congestive cardiac failure, particularly in the elderly. A review of transfusion outcome in patients with acute coronary artery syndromes revealed a greater mortality rate in transfusion recipients [140]. Another review suggested that treatment of mild to moderate anaemia resulted in increased mortality [141]. Also transplant recipient sensitisation may occur following transfusion resulting in longer transplant register waiting times and difficulty in finding a cross match negative donor. A study from Ireland looking at causes of sensitisation of potential allograft recipients showed that the level of sensitisation increased with the number of units of blood transfused and also demonstrated a direct relationship between degree of sensitisation and waiting time for transplantation [142]. Blood transfusions can induce antibodies to histocompatibility leukocyte antigens that can reduce the success of kidney transplantation; thus transfusions generally should be avoided in patients awaiting a renal transplant [141].

The use of ESAs can greatly reduce the need for red blood cell transfusions in patients with anaemia of CKD when target Hb concentrations are achieved and maintained [143, 144]. Since the introduction of ESAs and reduction in routine blood transfusion in anaemic patients with CKD sensitisation has markedly reduced [145]. With the advent of new immunosuppressant regimens after 1995, the use of pre-transplantation transfusion have been rendered largely obsolete. The K-DOQI anaemia guideline recommends that no single Hb concentration justifies or requires transfusion and the target Hb recommended for chronic anaemia management should not serve as a transfusion trigger. NICE agrees that there are clinical reasons to minimise blood transfusion in anaemia of CKD and if blood transfusion is essential the relevant haematology guidelines should be followed (e.g. the British Committee for Standards in Haematology (BCSH) guidelines www.bcshguidelines.com) [146]. In hospitalised patients who are haemodynamically stable, the need for transfusion is directed by symptoms and the Hb values. A value in CKD patients of <70 g/L or <80 g/L in post-operative surgical patients or pre-existing cardiac disease should prompt transfusion.

Hepatitis E virus (HEV) is a RNA virus and has 4 genotypes: the one commonly found in the UK is genotype 3. The most common route of infection in the UK is from eating raw or undercooked meat (particularly pork products) and shellfish; however, HEV can be transmitted via blood transfusion and solid organ transplantation. Incidence of HEV in the UK has been increasing considerably since 2011. It is likely that as many as 100,000 persons may suffer acute infections each year and that less than 1 in 100 will have any illness at all1. The majority of people who become infected with HEV have no symptoms and the infection clears completely within a couple of months. HEV may pose a risk of harm to immunocompromised patients who may be unable to clear the infection, which may then become persistent, potentially leading to chronic inflammation of the liver and cirrhosis. The Advisory Committee on the Safety of Blood, Tissues and Organs (SaBTO) recommends that immunocompromised /immunosuppressed patients should receive HEV negative blood components [147].

### Anaemia of CKD (Guideline 6.1)

#### Guideline 6.1 - post-transplantation Anaemia

We recommend that the treatment guidelines for anaemia in renal transplant patients should be similar to those for CKD patients not on dialysis (2B).

##### Rationale

Post transplantation Anaemia (PTA) is common [148–150]. Apart from the usual causes of anaemia due to CKD, renal transplant recipients have various unique factors predisposing to anaemia.

Factors causing PTA:

1. GFR: anaemia in transplant patients reflects the degree of GFR similar to other patients with CKD [150].
2. Immunosuppressive medications: Mycophenolate and azathioprine are myelosuppressive agents. Calcineurin inhibitors may cause anaemia by microangiopathic haemolysis [151–155]. OKT3 may also cause haemolytic uraemic syndrome (HUS) [156, 157]. Tacrolimus has also been associated with anaemia [158–160]. It may interfere with post erythropoietin receptor binding intracellular signalling and may occasionally cause HUS [160, 161].
3. Angiotensin converting enzyme (ACE) inhibitor and angiotensin receptor blocker (ARB) use: ACE inhibition has been linked with anaemia [150, 162]. Its pathogenesis is multifactorial and may include inhibition of endogenous EPO production, production of an erythropoiesis-inhibiting protein [163] and inhibition of angiotensin II mediated stimulation of erythrocyte precursors [164].
4. Antibiotic use: various common antibiotics may cause anaemia including trimethoprim-sulfamethoxazole.
5. Infections: viral infections such as cytomegalovirus and parvovirus B19 and antiviral agents such as ganciclovir may cause anaemia in transplant patients [165, 166].
6. Malignancy: malignancies including post-transplant lymphoproliferative disorder may result in anaemia.
7. Haemolytic anaemia: haemolytic anaemia may result from HUS or minor blood group incompatibility in transplant patients [167–169].
8. Rejection episodes: Acute rejection may cause reduced endogenous EPO production [170]. Severe vascular rejection may cause microangiopathy.
9. Chronic inflammation: Failing renal transplant causes a chronic inflammatory state resulting in EPO hypo-responsiveness.

##### Safety of ESA in transplant patients

A few early retrospective studies suggested increased incidence of delayed graft function in patients on ESA prior to transplantation [171, 172]. However Registry data has since shown reduced incidence of delayed graft function despite increasing use of ESA. It has also been shown that ESA use prior to renal transplantation does not reduce production of or response to endogenous EPO [173, 174]. Studies in the early post-transplant period did not show significant adverse events including delayed graft function or hypertension [175, 176]. Studies in the late transplant period have shown increased incidence of hypertension [177, 178]. ESAs, most probably, do not accelerate rate of graft function decline and one study suggested that correction of anaemia slowed the decline in allograft function [179].

In another prospective study that assessed the effect of correction of anaemia on progression of renal Insufficiency in transplant patients; 128 patients from 17 centres in France treated with ESA were randomised to full correction of anaemia (hemoglobin values13.0–15.0 g/dl, *n* = 63) versus partial correction of anemia (Hb value 10.5–11.5 g/dl, *n* = 62).This study found that in the group of patients with a haemoglobin level close to normal (~13 g/dL), the rate of decline of renal function was lower compared with the group of control patients, and the number of patients reaching end-stage renal disease and the number of graft failures was lower in this treatment group compared with the control group, suggesting that correcting anaemia in transplant patients reduces the rate of decrease of renal function and reduces the number of grafts lost [180].

##### Efficacy of ESA in transplant patients

Studies in the early post-transplant period have shown that ESA is effective in these patients, although the dose required may be higher than in pre-transplant period [175, 176]. Similarly studies in late post-transplant period have shown efficacy of ESA in these patients [177, 178, 181, 182].

## Lay summary

Anaemia is a commonly diagnosed complication among patients suffering with chronic kidney disease. If left untreated, it may affect patient quality of life. There are several causes for anaemia in this patient population. As the kidney function deteriorates, together with medications and dietary restrictions, patients may develop iron deficiency, resulting in reduction of iron supply to the bone marrow (which is the body organ responsible for the production of different blood elements). Chronic kidney disease patients may not be able to utilise their own body’s iron stores effectively and hence, many patients, particularly those receiving haemodialysis, may require additional iron treatment, usually provided by infusion.

With further weakening of kidney function, patients with chronic kidney disease may need additional treatment with a substance called erythropoietin which drives the bone marrow to produce its own blood. This substance, which is naturally produced by the kidneys, becomes relatively deficient in patients with chronic kidney disease. Any patients will eventually require treatment with erythropoietin or similar products that are given by injection.

Over the last few years, several iron and erythropoietin products have been licensed for treating anaemia in chronic kidney disease patients. In addition, several publications discussed the benefits of each treatment and possible risks associated with long term treatment. The current guidelines provide advice to health care professionals on how to screen chronic kidney disease patients for anaemia, which patients to investigate for other causes of anaemia, when and how to treat patients with different medications, how to ensure safe prescribing of treatment and how to diagnose and manage complications associated with anaemia and the drugs used for its treatment.
